# O-GlcNAcylation of ATG4B positively regulates autophagy by increasing its hydroxylase activity

**DOI:** 10.18632/oncotarget.11083

**Published:** 2016-08-05

**Authors:** Yoon Kyung Jo, Na Yeon Park, So Jung Park, Byung-Gyu Kim, Ji Hyun Shin, Doo Sin Jo, Dong-Jun Bae, Young-Ah Suh, Jeong Ho Chang, Eun Kyung Lee, Sang-Yeob Kim, Jin Cheon Kim, Dong-Hyung Cho

**Affiliations:** ^1^ Department of Gerontology, Graduate School of East-West Medical Science, Kyung Hee University, Yongin, South Korea; ^2^ Leading-edge Research Center for Drug Discovery and Development for Diabetes and Metabolic Disease, School of Medicine, Kyungpook National University Hospital, Daegu, South Korea; ^3^ Asan Institute for Life Sciences, Asan Medical Center, Seoul, South Korea; ^4^ Department of Biology Education, Kyungpook National University, Daegu, South Korea; ^5^ Department of Biochemistry, College of Medicine, Catholic University of Korea, Seoul, South Korea; ^6^ Department of Surgery, University of Ulsan College of Medicine, Asan Medical Center, Seoul, South Korea

**Keywords:** autophagy, ATG4B, *O*-GlcNAcylation, OGT, SH-SY5Y cells

## Abstract

Autophagy is a catabolic degradation process and maintains cellular homeostasis. And autophagy is activated in response to various stress conditions. Although *O*-GlcNAcylation functions a sensor for nutrient and stress, the relationship between *O*-GlcNAcylation and autophagy is largely unknown. Here, we identified that ATG4B is novel target for *O*-GlcNAcylation under metabolic stress condition. Treatment with PugNAc, an *O*-GlcNAcase inhibitor increased activation of autophagy in SH-SY5Y cells. Both bimolecular fluorescence complementation and immunoprecipitation assay indicated that OGT directly interacts with ATG4B in SH-SY5Y cells. We also found that the *O*-GlcNAcylated ATG4B was increased in autophagy activation conditions, and down-regulation of OGT reduces *O*-GlcNAcylation of ATG4B under low glucose condition. Furthermore, the proteolytic activity of ATG4B for LC3 cleavage was enhanced in PugNAc-treated cells. Taken together, these results imply that *O*-GlcNAcylation of ATG4B regulates autophagy activation by increasing its proteolytic activity under metabolic stress condition.

## INTRODUCTION

Autophagy functions under various stress conditions [1, 2]. Among the different stress-causing conditions, glucose deprivation is a significant source of stress to neuronal cells because of their high demand for glucose [3, 4]. Autophagy is regulated by multiple signaling pathways and autophagy-related proteins (ATGs) essentially control the process [1, 2]. Mammalian target of rapamycin (mTOR) is activated in response to various stimuli including amino acids, energy levels, oxygen, growth factors, and stress controlling metabolic homeostasis [5, 6]. Nutrient deficiency negatively regulates mTOR complex 1 (TORC1) which directly represses the ULK1 kinase complex [7, 8]. Autophagy is initiated with the formation of the phagophore, which is mediated by ULK1 and class 3-kinase complex [9]. Then elongation and maturation of autophagosomes are performed by two ubiquitin-like reactions [10]. In the first reaction, ATG7, ATG10, and the ATG5-ATG12-ATG6 complex elongate the pro-autophagosomal membrane [10]. The second process generates the protein microtubule-associated protein 1 light chain 3 (MAP1-LC3/LC3/Atg8), which mediates membrane tethering and hemifusion [11]. LC3 is produced as a precursor protein and generated LC3-I by the protease ATG4B, then it is conjugated with phosphatidylethanolamine (PE) in an ubiquitin-like reaction to generate LC3-II. Finally, LC3-II is specifically targeted to the elongating autophagosome membrane [12, 13]. Once formation of the autophagosome is completed, the autophagosome fuses with the lysosome. And the LC3-II on the cytoplasmic location of autolysosome is recycled after delipidation of PE, which is also performed by ATG4B [14].

Several transcription factors such as farnesoid X receptor (FXR), cAMP response element-binding protein (CREB), and transcription factor EB (TFEB) coordinately modulate the expression of ATG genes [15–18]. In addition, ATG gene expression is controlled by epigenetic changes [19, 20]. Autophagy is regulated not only by expression regulation but also by post-translational modifications (PTMs). PTMs play a critical role in the regulation of protein activity. Indeed, PTMs of ATG proteins, including phosphorylation, ubiquitination, lipidation, methylation, and acetylation have been reported [21]. For example, ULK1 is a kinase itself, and several protein kinases phosphorylate ULK1 at multiple sites to regulate initiation of autophagy [22–25]. In addition, ubiquitination/de-ubiquitination of ATG6/Beclin 1 and ULK1 also regulate autophagic pathways [26, 27]. Deacetylation of ATG5, ATG7, ATG8, and ATG12 by SIRT1 can initiate autophagy under starvation conditions [28]. Furthermore, lipidation of Atg8/LC3 is associated with the elongation and maturation of the autophagosome [29–31]. ATG4B plays critical roles in LC3 lipidation and processing as well [31, 32].

*O*-linked β-N-acetylglucosamine (*O*-GlcNAc) is an intercellular carbohydrate that dynamically modifies proteins, and *O*-GlcNAcylation is a process involving attachment of *O*-GlcNAc to the -OH group of Serine, Threonine, Tyrosine (Ser/Thr/Tyr) residues [33, 34]. Because of target residue specificity, crosstalk between *O*-GlcNAcylation and phosphorylation may regulate protein modification [35]. *O*-GlcNAcylation is known to be involved in a number of cellular events, including transcription, cell cycle, and several signal transductions [36, 37]. Unlike protein phosphorylation which is medicated by several hundred kinases, *O*-GlcNAcylation is mediated by *O*-GlcNAc transferase (OGT), whereas *O*-GlcNAcase (OGA) catalyzes hydrolytic cleavage of O-GlcNAc from proteins [33]. Thus, OGT and OGA are paired enzymes which are only responsible for regulation of attachment and detachment of *O*-GlcNAc. The expression level and activity of OGT is regulated by cellular metabolic stress condition [38, 39]. The enzymatic activity of OGT is closely related to the concentration of uridine diphospho-GlcNAc (UDP-GlcNAc), an immediate donor substrate for *O*-GlcNAcylation and the final product of hexosamine biosynthetic pathway (HBP) [40]. Upon HBP flux change, UDP-GlcNAc levels change rapidly and *O*-GlcNAcylation of many proteins is ultimately altered. In hyperglycemia, elevated HBP flux causes increased *O*-GlcNAcylation on specific insulin signaling molecules [34, 41]. In addition, various stresses increase glucose uptake and thus HBP flux and concomitantly increase UDP-GlcNAc concentrations, leading to a rapid global increase in *O*-GlcNAcylation [32, 43].

*O*-GlcNAcylation is a sensor for nutrients and stress that also highly influences autophagy activation [36, 43]. Nonetheless, the precise mechanism that coordinates autophagy regulation by *O*-GlcNAcylation remains unclear. In the present study, we identified ATG4B as a novel substrate for *O*-GlcNAcylation. Treatment with an OGA inhibitor increased autophagy flux. In addition, OGT directly bound to ATG4B and induces *O*-GlcNAcylation of ATG4B. Moreover, the protease activity of ATG4B was positively regulated by *O*-GlcNAcylation under low glucose condition. Taken together, these results suggested that *O*-GlcNAcylation of ATG4B regulates autophagy by increasing its protease activity.

## RESULTS

### PugNAc treatment induces activation of autophagy in SH-SY5Y cells

**Figure 1:**
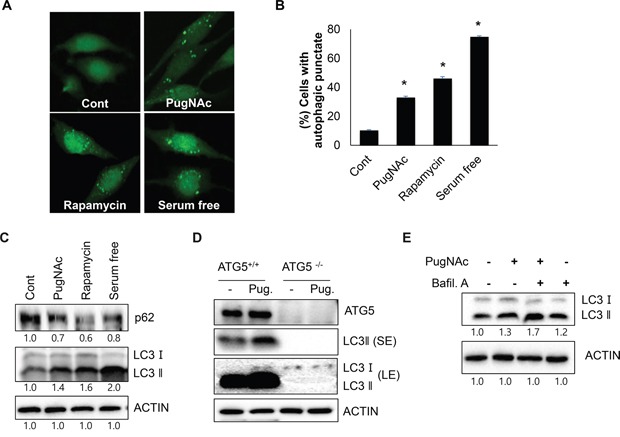
Treatment with PugNAc, an OGA inhibitor activates autophagy **A-C.** SH-SY5Y/LC3-GFP cells were treated with an OGA inhibitor (PugNAc, 100 μM), rapamycin (0.5 μM), or were incubated with in serum free condition for 24 h. Then, the cells were fixed and imaged LC3-GFP punctate with a fluorescent microscopy (A), or the cells with autophagic punctate were counted under a fluorescence microscopy (B). The cells were then harvested and analyzed by Western blotting with p62 and LC3 antibodies (C). **D.** Wild type MEF (+/+) and ATG5 knock out (−/−) MEF treated with PugNAc (Pug, 100 μM) for 24 h were harvested, and conversion of LC3 protein was analyzed by Western blotting (SE; short expose, LE; long expose). **E.** For autophagy flux assay, SH-SY5Y cells pre-treated with PugNAc (100 μM) for 24 h were additionally incubated with or without an autophagy inhibitor bafilomycine A1 (Bafil A, 0.1 μM) for 3 h. The cells were analyzed by Western blotting with LC3 antibody. Data are represented as the mean ± SEM (n > 3, and * p < 0.05 value).

Although O-GlcNAcylation has been reported to act as a stress sensor in metabolic signaling, the relationship between O-GlcNAcylation and autophagy is not well understood. To explore the effect of *O*-GlcNAcylation on autophagy, we examined the effect of PugNAc treatment on autophagy level. Because PugNAc is an OGA inhibitor, it functions as an *O*-GlcNAcylation inducer. SH-SY5Y cells stably expressing GFP-fused LC3 protein, an autophagy marker (SY5Y/GFP-LC3) were dose-dependently treated with PugNAc, and cells with LC3 punctate were observed under a fluoresce microscopy. As shown in Figure 1A and 1B, treatment with PugNAc strongly induced the formation of autophagic punctate dots by GFP-LC3 in SH-SY5Y cells (Figure 1A, 1B). Both the formation of punctate structures with GFP-fused LC3 protein and the increased production of LC3 II protein have been generally used as monitoring marker of autophagy activation [6]. In addition, p62 is a well-known substrate for autophagic degradation [6]. Thus, we further assessed the level of LC3 protein and p62. In accordance with this notion, treatment with PugNAc resulted in enhanced level of LC3 II as well as reduction of p62 level (Figure 1C). ATG5 is an essential regulator of autophagosome formation during autophagy. To examine the effect of blockage the autophagy activation in PugNAc-treated cells, wild type mouse embryo fibroblasts (WT MEF) and ATG5 knock out MEF (ATG5^−/−^ MEF) cells were treated with PugNAc, and then level of LC3 proteins was detected. Consistent with previous results, treatment of PugNAc induced autophagy in WT MEF cells, however it failed to activate autophagy in ATG5^−/−^ MEF cells (Figure 1D). Based on these result, we further examined autophagy flux in PugNAc-treated cells. Combined treatment PugNAc with a lysosome fusion blocker, bafilomycin A1, resulted in more accumulation of LC3 II protein than that of PugNAc alone (Figure 1E), suggesting that treatment with PugNAc induces the activation of autophagy in SH-SY5Y cells.

### OGT interacts with ATG4B in SH-SY5Y cells

**Figure 2:**
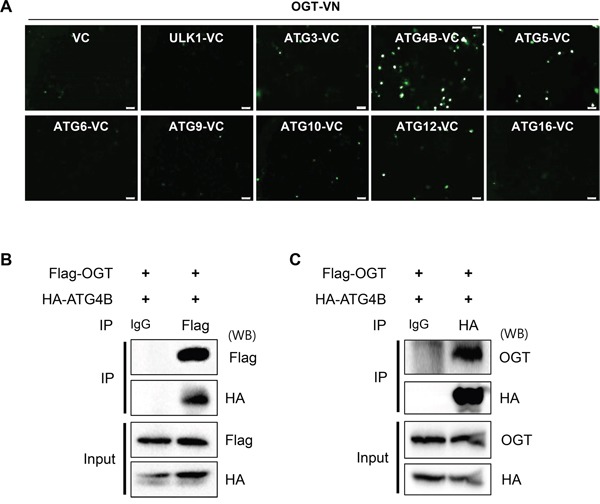
OGT directly binds to ATG4B in SH-SY5Y cells **A.** SH-SY5Y cells were transiently co-transfected with BiFC plasmids [(pFlag/OGT–VN173 (OGT-VN) and pMYC/ATG–VC155 (ATGx-VC)]. After 48 h, the fluorescence intensity of the cells was imaged by a fluorescence microscopy. **B.** SH-SY5Y cells stably expressing HA-tagged ATG4B (SY5Y/HA-ATG4B) were transiently transfected with pFlag-OGT. After 48 h transfection, the cells were harvested and subjected into immunoprecipitation (IP) assay with anti-Flag antibody tagged with agarose-bead or IgG antibody. And the immuno-precipitates were analyzed with Western blotting (WB) with indicated antibodies. **C.** SH-SY5Y/HA-ATG4B cells transfected with pFlag-OGT were subjected into IP assay with anti-HA antibody tagged with agarose-bead or IgG antibody. And the immuno-precipitates were further analyzed with Western blotting with indicated antibodies.

To understand the molecular mechanism of how *O*-GlcNAcylation regulates autophagy, we employed a bimolecular fluorescence complementation (BiFC) assay system, which is used for the detection of protein interaction in living cells [45]. We generated an OGT-fused N-terminal Venus BiFC vector, pFlag/OGT–VN17 (OGT-VN) and various ATG-fused C-terminal Venus BiFC vectors, pMYC/ATG–VC155 (ATGx-VC) vector. To examine OGT binding counterpart, SH-SY5Y cells were co-transfected with OGT and various ATG plasmids for fusion with Venus fluorescent protein fragments. As shown in Figure 2A, BiFC signal was efficiently increased in cells expressing OGT-VN and ATG4B-VC, but not in those expressing ULK1, ATG3, ATG5, ATG6, ATG9, ATG10, ATG12 or ATG16 protein, suggesting that overexpressed OGT interacts with ATG4B in SH-SY5Y cells (Figure 2A). Then, we confirmed the possibility of the interaction between OGT and ATG4B. SH-SY5Y cells overexpressing HA-tagged ATG4B and Flag-tagged OGT were prepared, and the OGT protein complex was precipitated to detect ATG4B. As shown in Figure 2B, the immunoprecipitation (IP) assay confirmed the interaction of OGT with ATG4B. Moreover, the result of reciprocal IP assay further indicated that OGT is associated with ATG4B in SH-SY5Y cells (Figure 2C).

### ATG4B is *O*-GlcNAcylated in PugNAc-treated cells

**Figure 3:**
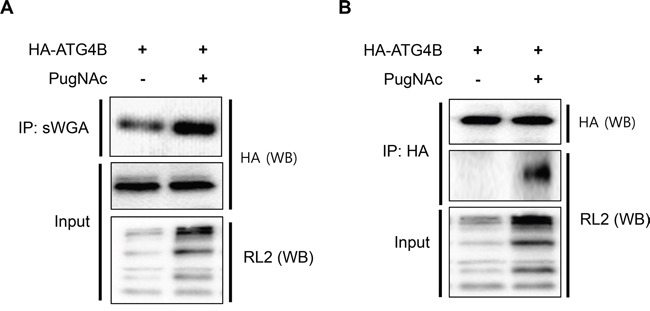
ATG4B is *O*-GlcNAcylated in PugNAc-treated cells **A.** SY5Y/HA-ATG4B cells were cultured in the presence or absence of PugNAc (100 μM). After 24 h, whole *O*-GlcNAcylated proteins in the cells were pull-down with sWGA-agarose antibody, and the precipitated sWGA-complex was analyzed with Western blotting (WB) with anti-HA antibody and RL2 antibody recognizing *O*-GlcNAc-protein. **B.** SY5Y/HA-ATG4B cells were treated with PugNAc (100 μM) for 24 h. The cells lysates were harvested and subjected to immunoprecipitation (IP) with anti-HA-agarose antibody. The *O*-GlcNAcylated protein was detected with anti-RL2 antibody. Enhanced *O*-GlcNAcylated proteins in PugNAc-treated cells (Input) were analyzed by immunoblotting with RL2 antibody.

We next examined whether ATG4B is a target of *O*-GlcNAcylation. To induce *O*-GlcNAcylation of proteins, SH-SY5Y cells overexpressing HA-tagged ATG4B (SY5Y/HA-ATG4B) were treated with PugNAc. Whole *O*-GlcNAcylated proteins were immunoprecipitated with sWGA antibody, which is widely used for detection and enrichment of *O*-GlcNAcylated proteins. *O*-GlcNAcylation of ATG4B was examined using the sWGA-immunoprecipitates and ATG4B was addressed. We confirmed increased *O*-GlcNAcylation byRL-2, an *O*-GlcNAc antibody in PugNAc-treated cells (Figure 3A). The IP assay indicated that ATG4B is modified by *O*-GlcNAcylation (Figure 3A). We additionally verified *O*-GlcNAcylation of ATG4B by reciprocal immunoprecipitation assay. The IP assay using anti-HA and *O*-GlcNAc antibodies indicated that ATG4B is modified by *O*-GlcNAc (Figure 3B). Then we attempted to map the target site for *O*-GlcNAcylation. To identify the *O*-GlcNAcylated residue, a mass spectrometric analysis was employed. HA-tagged ATG4B was purified with anti-HA-agarose bead from PugNAc-treated SY5Y/ATG4B cells and *O*-GlcNAcylated peptides were analyzed. The mass analysis revealed various putative candidate sites for *O*-GlcNAcylation of ATG4B including Thr5, Thr10, Ser23, Ser34, Thr37, Ser68, Thr70, Ser110, and Ser138 (Supplementary Figure S1). Based on the mass results, we generated several point mutations of ATG4B, and investigated *O*-GlcNAcylation. However, those mutants were still *O*-GlcNAcyled in PugNAc-treated cells (Supplementary Figure S2), suggesting that ATG4B might have multiple target restudies for *O*-GlcNAcylation. Although target site was not identified, these results suggested that ATG4B is *O*-GlcNAcylated in PugNAc-treated cells.

### *O*-GlcNAcylation of ATG4B is enhanced in low glucose condition

**Figure 4:**
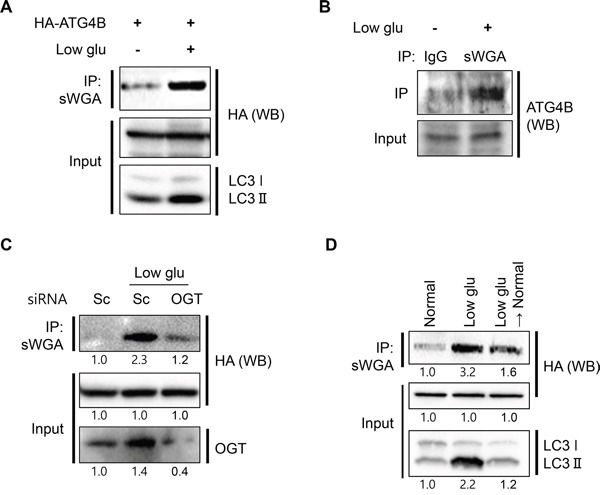
Low glucose condition promotes *O*-GlcNAcylation of ATG4B **A.** SH-SY5Y/HA-ATG4B cells were cultured in normal (4.5 mg/ml glucose) or low glucose condition (Low glu, 1 mg/ml glucose). After 24 h, total *O*-GlcNAc proteins were pull-down using sWGA-agarose antibody and the immune-complex was analyzed with Western blotting (WB) with anti-HA antibody. And the input lysates were further detected with anti-HA and anti-LC3 antibodies. **B.** For endo-IP assay, SH-SY5Y cells were incubated in normal or low glucose condition. And whole *O*-GlcNAc proteins were pull-down using sWGA-agarose antibody or IgG antibody. The immune-precipitates were further assessed with Western blotting with anti ATG4B antibody. **C.** SY5Y/HA-ATG4B cells transiently transfected with either scrambled siRNA (Sc) or siRNA against OGT were cultured in low glucose condition for 24 h. Then the cell lysates were analyzed by Western blotting with anti-HA antibody after immunoprecipitation with anti-sWGA-agarose antibody. Knock down of OGT protein by siRNA was confirmed by immunoblotting with anti-OGT antibody. **D.** SH-SY5Y/ATG4B cells were incubated in low glucose (1 mg/ml glucose) for 24 h. And the cells were recovered with normal high glucose media (4.5 mg/ml glucose) or not for additional 3 days. *O*-GlcNAc proteins were pull-down using sWGA-agarose antibody and immuneblotted with anti-HA antibody.

*O*-GlcNAcylation functions as a nutrient and stress sensor that highly influences autophagy activation. For example, low glucose condition upregulates both expression level and activity of OGT [38, 39]. To clarify the role of nutrient sensing in the regulation of *O*-GlcNAcylation of ATG4B, we investigated *O*-GlcNAcylated ATG4B in the context of glucose deprivation. SY5Y/HA-ATG4B cells were cultured in low glucose condition (1 mg/ml glucose) and *O*-GlcNAcylated ATG4B was addressed by IP assay. As shown in Figure 4A, deprivation of glucose strongly increased the level of *O*-GlcNAcylated ATG4B as well as activation of autophagy (Figure 4A). Then we confirmed *O*-GlcNAcyation of endogenous ATG4B in glucose-reduced SH-SY5Y cells. Western blot analysis with anti-ATG4B antibody following IP assay with anti-sWGA antibody in glucose-reduced situation showed that endogenous level of *O*-GlcNAcylated ATG4B was also enhanced in glucose-reduced SH-SY5Y cells (Figure 4B). In order to examine the effect of OGT on *O*-GlcNAcylation of ATG4B, we depleted OGT by its siRNA. SY5Y/HA-ATG4B cells were transfected with either control or specific siRNA against OGT, and the cells were incubated in low glucose condition. Concordant with our hypothesis, suppression of OGT resulted in reduction of *O*-GlcNAcylated ATG4B during glucose deprivation (Figure 4C). Then we further examined the effect of change of glucose concentration. SY5Y/HA-ATG4B cells cultured in low glucose (1 mg/ml glucose) were recovered with high glucose media or not, and then IP assay was performed. As shown in Figure 4D, change of culture media form low to high concentration of glucose decreased *O*-GlcNAc-ATG4B (Figure 4D). Taken together, these results suggested that *O*-GlcNAcylation of ATG4B is increased in low glucose condition.

### *O*-GlcNAcylation of ATG4B enhances its proteolytic activity in SH-SY5Y cells

**Figure 5:**
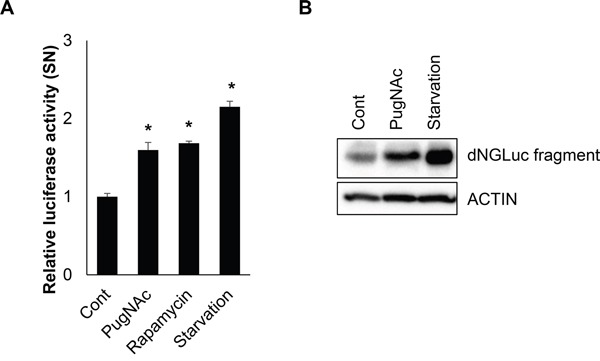
*O*-GlcNAcylation of ATG4B enhances proteolytic activity of ATG4B **A.** SH-SY5Y cells were treated with an OGA inhibitor (PugNAc, 100 μM), rapamycin (0.5 μM), or were incubated in a serum starvation condition. After 24 h treatment, the supernatant (SN) was collected prior to analysis of a luciferase activity, and relative luciferase activity was measure. **B.** SH-SY5Y cells transiently transfected with pEAK12-ACTIN-LC3-dNGLUC were treated with PugNAc (100 μM) or incubated in a serum starvation condition. And the cells were further treated with Brefeldin A (10 μg/ml) for 6 h to block secretion of cleaved dNGLUC form before harvesting cells. Then the cell lysates were analyzed by Western blotting with anti-luciferase antibody. Data are represented as the mean ± SEM (n > 3, and * p < 0.05 value).

Although more than 40 ATG proteins have been identified in yeast, only ATG4 shows protease activity. LC3 is known to be an important substrate for ATG4B in generation of autophagosomes. Therefore, we investigated the effects of *O*-GlcNAcylation on ATG4B. In order to evaluate the function of *O*-GlcNAcylated ATG4B, we employed an assay system which had already been validated for ATG4B activity. Ketteler *et al*. previously introduced a luciferase-based assay that specifically measures the induction of autophagy by monitoring proteolytic activity of ATG4B [46, 47]. And it is suitable for cell-based *in vivo* analysis of ATG4B activity. Based on this assay system, SH-SY5Y cells expressing pEAK12-Actin-LC3-dNGLUC were treated with either PugNAc or rapamycin, or the cells were incubated in serum starved condition. Then, Gaussia luciferase (GLUC) activity was measured. Interestingly, luciferase activity was significantly increased in PugNAc-treated cells as well as in starved cells, suggesting that *O*-GlcNAcylation of ATG4B increased cleavage of linked LC3 protein in SH-SY5Y cells (Figure 5A). Furthermore, we examined the cleavage product of Actin-LC3-dNGLUC in the cells. Consistent with previous results, cleaved fragment by ATG4B was also markedly increased in PugNAc-treated cells (Figure 5B). These results suggested that *O*-GlcNAcylation of ATG4B enhances its proteolytic activity.

## DISCUSSION

Accumulating evidence indicates that PTM of ATG proteins affords significant flexibility for the modulation of autophagy activity [21]. Furthermore, several studies have suggested that *O*-GlcNAcylation regulates autophagy activation as a PTM manner [48–51]. Unlike typical *O*-and *N*-linked glycosylation which is modified in the endoplasmic reticulum and golgi apparatus, *O*-GlcNAcylation is occurs on cytoplasm and nucleus. To date more than 1000 proteins have been identified as *O*-GlcNAc targets [21].

Regarding with autophagy, it was recently reported that SNAP29, a SNARE protein dependently regulates maturation of autophagosomes by *O*-GlcNAcylation in *C. elegans* [48]. Both mTORC1 regulators such as AKT and AMPK, and Bcl-2 protein are direct targets for *O*-GlcNAcylation [49, 50]. Moreover, Marsh *et al*. showed that Beclin 1 is *O*-GlcNAcylated in cardiomyocytes, and autophagy is impaired in cardiomyocytes from diabetes mice [51]. Up-regulation of Beclin 1 by glucose deprivation was abolished by treatment with PugNAc in cardiomyocytes [51]. These reports strongly support for an important role of *O*-GlcNAcylation in autophagy. Nonetheless, the precise mechanism by which autophagy is regulated by *O*-GlcNAcylation remained largely unclear. In this study, we identified ATG4B as a novel substrate for *O*-GlcNAcylation. Yeast expresses a single gene encoding ATG4 protein, whereas there are four alternative splicing variants of ATG4 in mammals (ATG4A, 4B, 4C and 4D) [52]. Previous studies suggested that ATG4B is a universal regulator of LC3 in mammalian cells, but that other ATG4 family members may be specific for individual Atg8 paralogues [53, 54]. Therefore, further evaluation for *O*-GlcNAcylation by other ATG4 splicing variants could explain specific regulation of the proteins. Although Beclin 1 was reported as a target for *O*-GlcNAcylation, we could not detect *O*-GlcNAcylated Beclin 1. However, we also found that ATG7 as well as ATG4B could be *O*-GlcNAcylated from IP assay (data not shown). In addition, our BiFC assay indicated that interaction between ATG4B and OGT was higher than that of Beclin 1/ATG6 (Figure 2). These results implicate that *O*-GlcNAcylation can regulate autophagy by modulating various target proteins under different circumstances. Therefore, further investigations for the possibility of *O*-GlcNAcylation with several ATG proteins are remained to be elucidated. To date, numerous proteins have been identified as targets for *O*-GlcNAcylation. Some PTMs, such as phosphorylation, target a recognition motif. However, *O*-GlcNAcylation is not associated with a clear consensus motif. To identify a target region for *O*-GlcNAcylation on ATG4B, we performed a mass spectrometric analysis. Mass analysis revealed that various residues could be affected by *O*-GlcNAcylation. Based on the results, we generated ATG4B mutants with several putative candidate sites, and investigated the effect on *O*-GlcNAc modification. Unfortunately, the modification of those mutants still occurred. It is possible that there may be multiple target sites on ATG4B for *O*-GlcNAc modification or there is another target site for *O*-GlcNAc. Since we were not able to verify target residues in this study, further studies for identification of target residue should be performed.

Both *O*-GlcNAcylation and phosphorylation share target residues, Ser, Thr, and Tyr as the modification site, and recent omics data proposed that the interplay between *O*-GlcNAcylation and phosphorylation is more complex and comprehensive than previously anticipated [55]. Interestingly, most of *O*-GlcNAc modified proteins are also known phosphoproteins, suggesting that *O*-GlcNAc modification in proteins is intimately associated with phosphorylation [56]. Recently, Yang *et al*, showed that ATG4B can be phosphorylated and that phosphorylation negatively regulates its activity [57]. Phosphorylation of ATG4B at Ser383 and Ser392 is enhanced under starvation condition. However, they did not suggested inducer kinase for ATG4B phosphorylation [57]. In this study, we found that *O*-GlcNacylation of ATG4B is increased in metabolic stress condition (Figure 4). *O*-GlcNAc and phosphorylation in some cases have a reciprocal relationship where they compete for the same residue or adjacent sites. Therefore, further studies for implication of *O*-GlcNAcylation and phosphorylation on ATG4B will be helpful to understand regulation mechanism of autophagy.

During ischemic conditions, the brain is usually deprived of glucose, resulting in damage to the brain [58]. Although the direct effects of glucose deprivation on brain function are not clearly understood, the major symptom of Alzheimer's disease and dementia are associated with ischemia [59]. Autophagy is linked to cell survival and death, and we showed that *O*-GlcNAcylation of ATG4B is an important step in autophagy activation. Therefore, the further studies on the regulation of *O*-GlcNAcylation will be helpful to understand the role of autophagy in pathophysiological conditions including metabolic diseases and neurodegenerative diseases.

## MATERIALS AND METHODS

### Cell culture and stable transfection

SH-SY5Y cells were obtained from the American Type Culture Collection (ATCC, Manassas VA, USA). Wild type mouse embryonic fibroblast (MEF) and ATG5 knock out MEF cells were kindly provided by Dr. Noboru Mizhushima (Tokyo University, Japan) [60]. Cells were cultured at 37°C in a 5% CO_2_ incubator and maintained in DMEM containing 10% FBS and 1% penicillin/streptomycin (Invitrogen, Carlsbad, CA, USA). To generate the stable cell line SH-SY5Y cells were transfected with either pEGFP-LC3 or pHA-ATG4B using Lipofectamine 2000 (Invitrogen) according to manufacturer's protocol. Stable transfectants were selected by G418 (1 mg/ml) for 10 days under fluorescence microscope or by Western blotting with anti-HA antibody.

### Reagents

The expression plasmid GFP-LC3 and Flag-OGT were kind gift from Dr. Noboru Mizushima (Tokyo University, Japan) and Dr. Jin Won Cho (Yonsei University, S. Korea). pBiFC vector was provide from Dr. Yong-Keun Jung (SNU, S. Korea). PugNAc (#3384) was purchased from TOCRIS ((Bristol, UK). Bafilomycin A1 (B1793) and Brefeldin A (B5936) were purchased from Sigma Aldrich (St. Louis, MO, USA). cDNAs encoding the full open reading frame of ATG4B was subcloned into the pcDNA3-HA (pHA-ATG4B). Various ATG4B mutants were generated using a Site-Directed Mutagenesis kit (Intron, S.Korea) and the mutation was confirmed by DNA sequencing analysis.

### Western blotting

For immunoblotting, cells were harvested using a lysis buffer (20 mM Tris-HCl (pH 7.5), 150 mM sodium chloride, 1 mM Na_2_EDTA, 1% Triton, 2.5 mM sodium pyrophosphate, 1 mM β-glycerophosphate, and protease inhibitor mixture). Samples separated by SDS PAGE were transferred to PVDF membranes. The membranes were blocked in 4% skim milk. The membranes were immunoblotted with the following antibodies: anti-HA (Santa cruz (sc-7392), Santa Cruz, CA, USA); anti-Flag (Cell signaling (#2368); Beverly, MA, USA), anti-ATG4B (Cell signaling, #13507); anti-LC3 (Novus (NB100-2220) Littleton, CO, USA); anti-ATG5 (Abcam (ab54033), Cambridge, UK); anti-*O*-GlcNAc (Abcam, ab2739), anti-OGT (Abcam, ac97274); and anti-GLUC (New England BioLabs, (E8023S), Ipswich, MA, USA); and anti-Actin (Milipore (#MAB1501), Temecula, CA, USA). For protein detection, the membranes were incubated with HRP-conjugated secondary antibodies and signals were detected with EzWestLumi Plus (ATTO, Tokyo, Japan).

### Immunoprecipitation

Cells were homogenized in RIPA buffer (50 mM Tris-HCl (pH 7.5), 150 mM sodium chloride, 0.5% sodium deoxycholate, 1% triton X-100, 0.1% SDS, and 2 mM EDTA, and protease inhibitors). Cell lysates were pull down with anti-sWGA-agarose antibody (agarose attached succinylated wheat germ agglutinin, Vector Laboratories, AL-1023, AL-1023), Burlingame, CA, USA), anti-HA-agarose antibody (Santa cruz, sc-3792) or anti-Flag antibody (Cell signaling, #2368) with protein G plus/Protein A Agarose suspension (Calbiochem, #IP05, Billerica, MA, USA). After overnight incubation, sWGA and HA-conjugated samples were washed with RIPA buffer. Then the lysates were analyzed by Western blot analysis with each antibody.

### Bimolecular fluorescence complementation (BiFC) assay

For BiFC assay, OGT cDNA was subcloned into a Flag-tagged N-terminal Venus plasmid (pBiFC/Flag-VN173) and cDNA coding for ULK1, ATG3, ATG4B, ATG5, ATG6, ATG9, ATG10, ATG12, or ATG16 was subcloned into a MYC-tagged C-terminal Venus vector (pBiFC/MYC-VC155). BiFC assay was performed as previously described [42, 61]. Briefly, SH-SY5Y cells were cotrasnsfected with OGT fused to the N-terminus (OGT-VN) and each ATG was fused to the C-terminus (ATGs-VC) fragment of Venus. After 48h, fluorescence intensity resulting from dimerization of the Venus vectors, OGT-VN and ATGs-VC fusion protein was captured using a fluorescence microscope [Olympus, IX71].

### Mass spectrometric analysis

After in gel digestion, tryptic peptides are subsequently separated by online reversed-phase chromatography for each run using a Thermo Scientific Eazy nano LC II autosampler with a reversed-phase peptide trap EASY-Column and a reversed-phase analytical EASY-Column. Electrospray ionization was subsequently performed using a 30 μm (i.d.) nano-bore stainless steel online emitter (Thermo-Scientific) and a voltage set at 2.6 V. The chromatography system was coupled on-line with an LTQ Velos Orbitrap mass spectrometer. Protein identification was accomplished utilizing the Proteome Discoverer v1.3 database search engine (Thermo-Scientific) and searches were performed against in house ATG4B isoforms DB FASTA database. A fragment mass tolerance of 0.8 Da, peptide mass tolerance of 25 ppm, and maximum missed cleavage of 2 was set. Result filters was performed with peptide rank (Maximum rank: 1), peptides number per protein (Minimal number of peptides: 2, Count only rank 1 peptides: True, Count peptide only in top scored proteins: True) and Charge State versus Score. The Carbamidomethylation (+57.021 Da) of cysteine (C) is set as a Static Modification, and the following variable modifications were allowed: Oxidation/+15.99492 Da (M), HexNAc/+203.07937 Da (S, T), deamidated / +0.984 Da (N, Q).

### Luciferase activity assay

Cellular ATG4B activity was measured using an N-terminal deleted form of Gaussia luciferase (dNGLUC) as previously reported by Ketter, *et al*. [46, 47]. Briefly, SH-SY5Y cells were transiently transfected with pEAK12-ACTIN-dGLUC or pEAK12-ACTIN-LC3-dNGLUC by using Lipofectmine 2000 (Invitrogen, Carlsbad, CA, USA) according to the manufacture's protocol. After 24 h, the medium was replaced and the cells were further treated with PugNAc, and then the luciferase assay was performed according to the manufacturer's instructions for the dual luciferase reporter assay system (Promega, Fitchburg, WI, USA).

### Statistical analysis

Data were obtained from least three independent experiments, and presented as means ± S.E.M. Statistical evaluation of the results was performed with one-way ANOVA. Data represent ± standard error of the mean (S.E.M.) from more than three independent experiments, n>3).

## SUPPLEMENTARY MATERIALS FIGURES


