# Supplementary material for: O-GlcNAcylation of ATG4B positively regulates autophagy by increasing its hydroxylase activity
Source: Oncotarget. 2016 Aug 5;7(35):57186–96. doi: 10.18632/oncotarget.11083 (PMC5302982; doi:10.18632/oncotarget.11083)
Supplement: Supplementary file 1 [file oncotarget-07-57186-s001.pdf]

O-GlcNAcylation of ATG4B positively regulates autophagy by increasing its hydroxylase activity

SUPPLEMENTARY FIGURES

A

Putative candidate sites (identified from MS analysis)	Sites
MDAATLTYDTLRFAEFEDFPETSEPVWILGR	Thr5, Thr10, Ser23
KYSIFTEK	Ser34, Thr37
KNFPAIGGTGPTSDTGWGCMLR	Ser68, Thr70
RQPDSYFSVLNAFIDR	Ser110
SIGQWYGPNTVAQVLK	Ser138

B

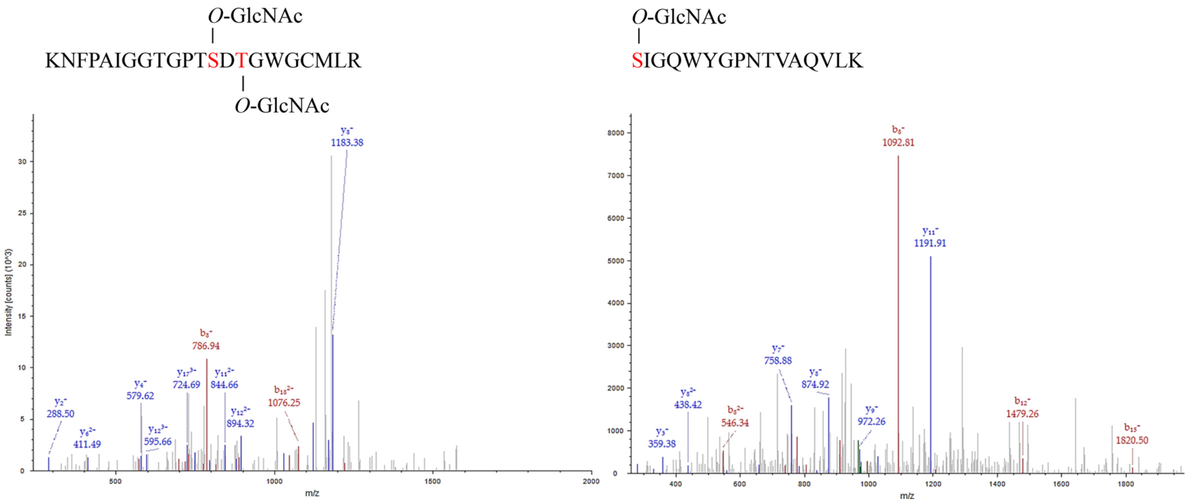

Supplementary Figure S1: Mass spectrometric analysis of ATG4B protein. A. List of putative candidate peptides of *O*-GlcNAacylation of ATB4B from mass spectrometric analysis. **B.** MS spectrum for the ATG4B fragmented peptide. SY5Y/HA-ATG4B cells were treated with PugNAc (100 µM) for 24 h. HA-tagged ATG4B protein was purified by immunoprecipitation and subjected into mass spectrometric analysis described in Material and Method.

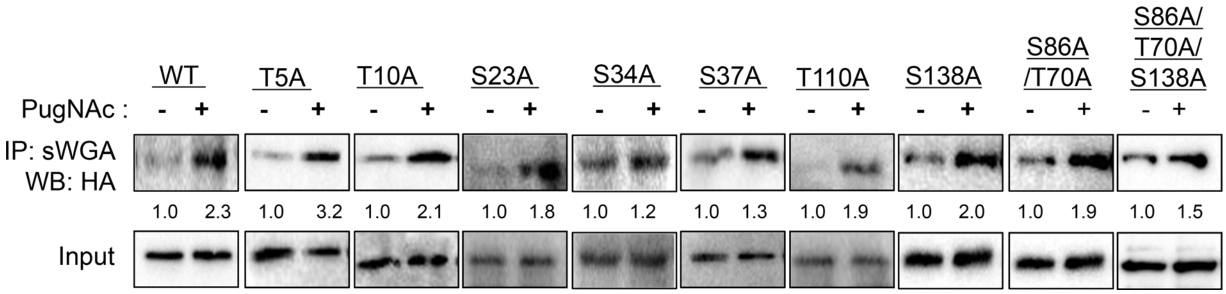

Supplementary Figure S2: Treatment with PugNAc, an OGA inhibitor activates autophagy. Wild type ATG4B (WT) and its mutants were overexpressed in SH-SY5Y cells, and the cells were treated with PugNAc (100 µM) for 24h. *O*-GlcNAcylated proteins in the cells were pull-down with sWGA-agarose antibody, and the precipitated sWGA-complex was analyzed with Western blotting with anti-HA antibody.
